# Cognitive Symptoms Across the Menopause Transition Are Not Explained by Acute or Chronic Stress

**DOI:** 10.1093/geroni/igaf122.2382

**Published:** 2025-12-31

**Authors:** Kavya Jhaveri, Teresa Facchetti, Sandra Rizer, Jadira Cuevas, Sarah Tom, Jillian Joyce, Mary Rosser, Stephanie Cosentino

**Affiliations:** Columbia University Irving Medical Center, New York, New York, United States; Columbia University Irving Medical Center, New York, New York, United States; Columbia University Irving Medical Center, New York, New York, United States; Columbia University Irving Medical Center, New York, New York, United States; Columbia University Irving Medical Center, New York, New York, United States; University of Southern California, Los Angeles, California, United States; Columbia University Irving Medical Center, New York, New York, United States; Columbia University Irving Medical Center, New York, New York, United States

## Abstract

Subjective cognitive difficulties are increasingly recognized as an early marker of Alzheimer’s disease. Cognitive complaints in women are often attributed to elevated stress, particularly during the menopausal transition. However, physiological changes during menopause may impact cognition. Here, we hypothesized that subjective cognitive difficulties would predict objective cognition independently of acute or chronic stress — acknowledging that while stress significantly impacts cognition, it does not fully explain the relationship between subjective and objective cognition. 112 women aged 45 + (mean age = 61.9, SD = 10.1, range=45-85) at different stages of menopause participated in the study. Subjective cognition was measured with a 6-item scale (SCoPE). The Linus Health Core Cognitive Evaluation, a digital cognitive health assessment, measured objective cognition (Digital Clock and Recall) and acute stress (“In the last month, there has been a significant increase in stress in my life”). Chronic stress was measured by the Ongoing Chronic Stressors Questionnaire (OCSQ). Linear regression showed SCoPE negatively predicted DCR (B=-.596, p=.008). Women with subjective complaints (N = 62) reported higher acute (Mann-Whitney U test: p = 0.043, U = 1251.0, Z=-2.024) and chronic stress (Mann-Whitney U test: p=.045, U = 1210.5, Z=-.2003) than those without (N = 50). In multiple regression adjusting for stress, SCoPE emerged as an independent predictor of DCR (B=-.564, p=.016) while stress was non-significant (OCSQ: B=-.031, p=.317; LHQ Q4: B=.043, p=.852). These findings suggest that while stress is elevated with subjective cognitive complaints, it does not fully account for their objective cognitive deficits, highlighting the need for multifaceted approaches to assess and intervene during menopausal transition to reduce dementia risk.

